# GAT-FD: An integrated MATLAB toolbox for graph theoretical analysis of task-related functional dynamics

**DOI:** 10.1371/journal.pone.0267456

**Published:** 2022-04-21

**Authors:** Meng Cao, Ziyan Wu, Xiaobo Li

**Affiliations:** 1 Department of Biomedical Engineering, New Jersey Institute of Technology, Newark, New Jersey, United States of America; 2 Department of Electrical and Computer Engineering, New Jersey Institute of Technology, Newark, New Jersey, United States of America; Beijing Normal University, CHINA

## Abstract

Functional connectivity has been demonstrated to be varying over time during sensory and cognitive processes. Quantitative examinations of such variations can significantly advance our understanding on large-scale functional organizations and their topological dynamics that support normal brain functional connectome and can be altered in individuals with brain disorders. However, toolboxes that integrate the complete functions for analyzing task-related brain functional connectivity, functional network topological properties, and their dynamics, are still lacking. The current study has developed a MATLAB toolbox, the Graph Theoretical Analysis of Task-Related Functional Dynamics (GAT-FD), which consists of four modules for sliding-window analyses, temporal mask generation, estimations of network properties and dynamics, and result display, respectively. All the involved functions have been tested and validated using functional magnetic resonance imaging data collected from human subjects when performing a block-designed task. The results demonstrated that the GAT-FD allows for effective and quantitative evaluations of the functional network properties and their dynamics during the task period. As an open-source and user-friendly package, the GAT-FD and its detailed user manual are freely available at https://www.nitrc.org/projects/gat_fd and https://centers.njit.edu/cnnl/gat_fd/.

## Introduction

Functional connectivity (FC), which quantifies temporal dependencies among spatially separated brain regions, has been highlighted as a sensitive and robust measurement in functional magnetic resonance imaging (fMRI) for understanding the topological organization of functional brain networks during sensory and cognitive processes and at resting-state [1, 2]. Typically, FC was evaluated in a “static” sense, which utilizes the entire scan duration for the connectivity calculation. Recently, accumulative evidence has suggested the temporally varying pattern of FC, referred to as dynamic FC, which can provide us a novel approach to depicting the non-stationarity of functional brain communications [3–5].

Currently, sliding-window-based techniques are commonly implemented for estimating FC dynamics [6–9]. Such approach applies an N-volume moving window along the time domain to estimate the pair-wise FCs based on signals of the current and previous N volumes. It thus generates a series of consecutive FC metrics, depicting the dynamically varying FC and topological organizations of the functional brain network.

To date, several toolkits have been developed for analyzing dynamic FC by using the sliding-window approach. The GIFT (http://mialab.mrn.org/software/gift/) is one of such toolkits, which has been commonly utilized in fMRI data [10–12]. The GIFT implements the K-means clustering method to effectively detect region-of-interest (ROI)-based stable brain states within a group of subjects when there is no external stimulus information, which makes it robust in evaluating ROI-based FC dynamics during resting-state [13, 14]. The DynaConn (http://www.drsakoglu.com/p/dynaconndfctoolbox.html), built based on GIFT toolbox’s functions, adds statistical analysis, task modulation analysis, and display options in analyzing the functional dynamics [15]. The DynamicBC (https://www.nitrc.org/projects/dynamicbc/) is another commonly implemented toolkit that calculates between-voxels, between-ROIs, and ROI-to-the reminder of whole brain FC dynamics in the temporal domain [16]. Compared to the GIFT, DynamicBC is more flexible in estimating ROI-based FC dynamics. The CONN (https://web.conn-toolbox.org/) is also frequently utilized for sliding window-based analysis of FC dynamics [17]. Relative to the GIFT and DynamicBC, CONN is a more comprehensive toolbox that provides preprocessing, static connectivity analysis, between-ROI dynamic variability analysis, and dynamic independent component analysis. The DyConPro (https://github.com/tobiamj/DyConPro) implements parallel factor analysis on dynamic connectivity signals to identify subnetworks that are related to individual differences [18]. Although these existing toolboxes have made it possible to estimate regional or between-ROI FC dynamics in fMRI data, they all lack the capacity in further assessing the temporal dynamics of the systems-level topological properties associated with the functional brain network during a cognitive task.

In this study, we introduce an open-source and user-friendly MATLAB toolbox, the Graph Theoretical Analysis of Task-Related Functional Dynamics (GAT-FD), which we have developed to integrate the complete pipelines for estimating the task-related dynamic brain FC and quantifying the topological dynamics and its statistical property of the functional brain network. This toolkit and the user manual with detailed explanations of each function and step-by-step instructions for implementations are freely available at https://www.nitrc.org/projects/gat_fd and https://centers.njit.edu/cnnl/gat_fd/.

## Methods

### Overview

**Fig 1:**
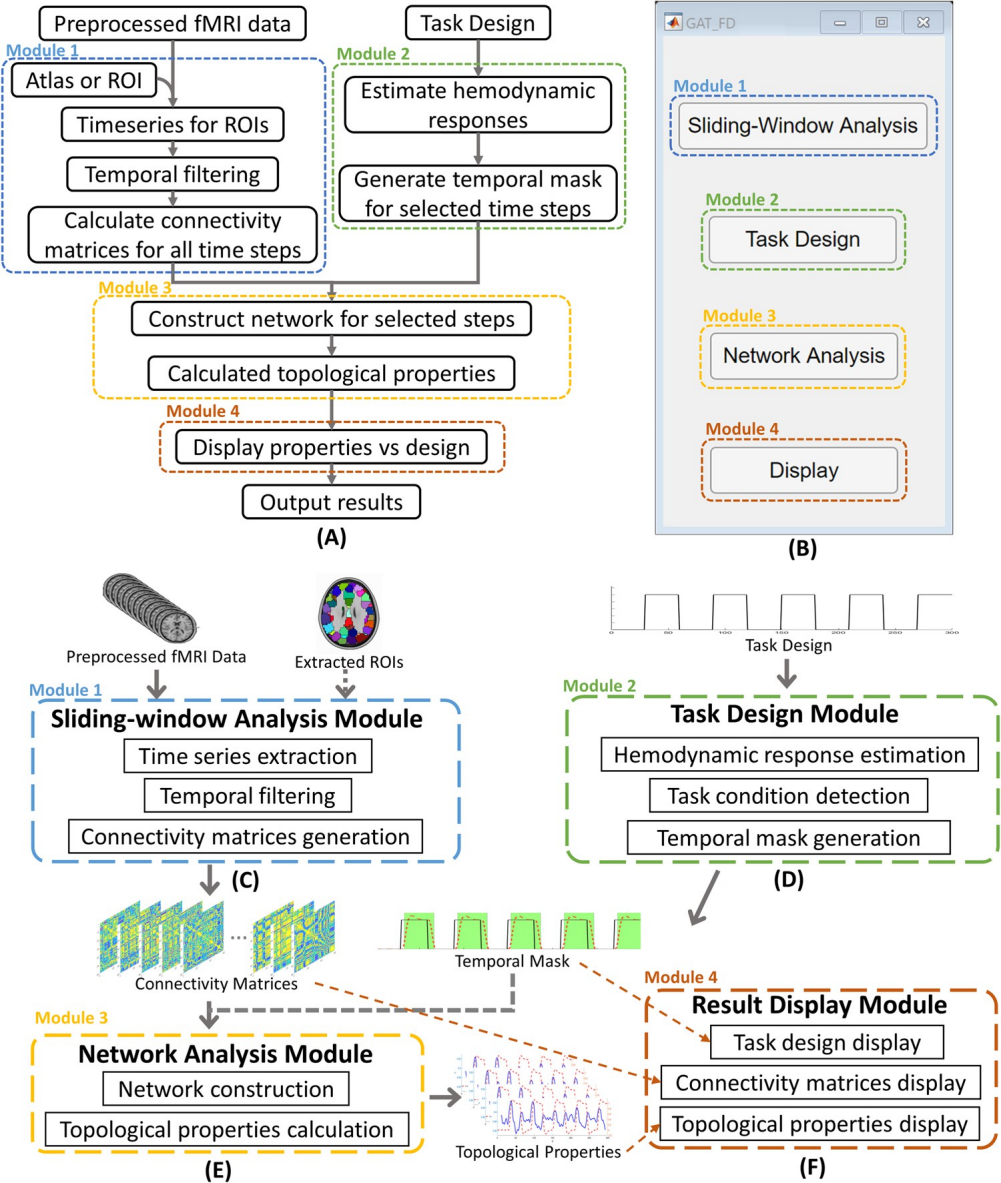
The four function modules of the GAT-FD toolbox. (A) A flowchart for analyzing functional network dynamics using GAT-FD toolbox. (B) The main user interface. (C) The sliding-window analysis module. This module generates connectivity matrices on the basis of specified sliding-window parameters. (D) The task design module. This module generates temporal inclusive mask according to the task design. (E) The network analysis module. This module calculates the topological properties for each connectivity matrix. (F) Result display module. This module provides visualization of the results from all other modules. fMRI: functional magnetic resonance imaging. ROIs: regions-of-interest. Note: Required steps are indicated using solid arrows. Optional steps are indicated using dashed arrows.

The GAT-FD toolbox provides a graphical user interface for characterizing the functional network dynamics in task-related fMRI data, based on the graph theoretical techniques. It was developed in MATLAB (Mathwork, Inc.) version 2019b and has been tested with MATLAB version 2016b to version 2019b. The GAT-FD is organized into four modules (Fig 1A and 1B) for sliding-window analysis (Fig 1C), task-specific temporal mask generation (Fig 1D), estimations of network properties (Fig 1E), and result display (Fig 1F), respectively. First, the sliding-window analysis module takes the preprocessed fMRI data and generates connectivity matrices based on selected atlas and sliding-window parameters. Next, the task design module creates temporal mask for dynamic analysis using task design information. Then, the network analysis module calculates the network properties for the time steps marked by the temporal mask. Last, the result display module plots the results for visual checking.

### Inputs

The GAT-FD toolbox works with pre-processed fMRI data. Properly pre-processed images can effectively minimize the falsely discovered dynamics caused by motion artifacts and undesired physiological fluctuations. The inputs are 4-dimonsional fMRI data in uncompressed or compressed NIfTI format (e.g., *.nii or *.nii.gz). All build-in atlases are in Montreal Neurological Institute (MNI) space. Therefore, all input data are required to be transformed from individual imaging space to MNI space, if the user is intended to use any of the build-in atlas. The toolbox also provides an option for the user to import MAT-format input files with a matrix containing the time series of each ROI, which allows extra flexibility in temporal processing.

### Sliding-window analysis and FC matrix construction

**Fig 2:**
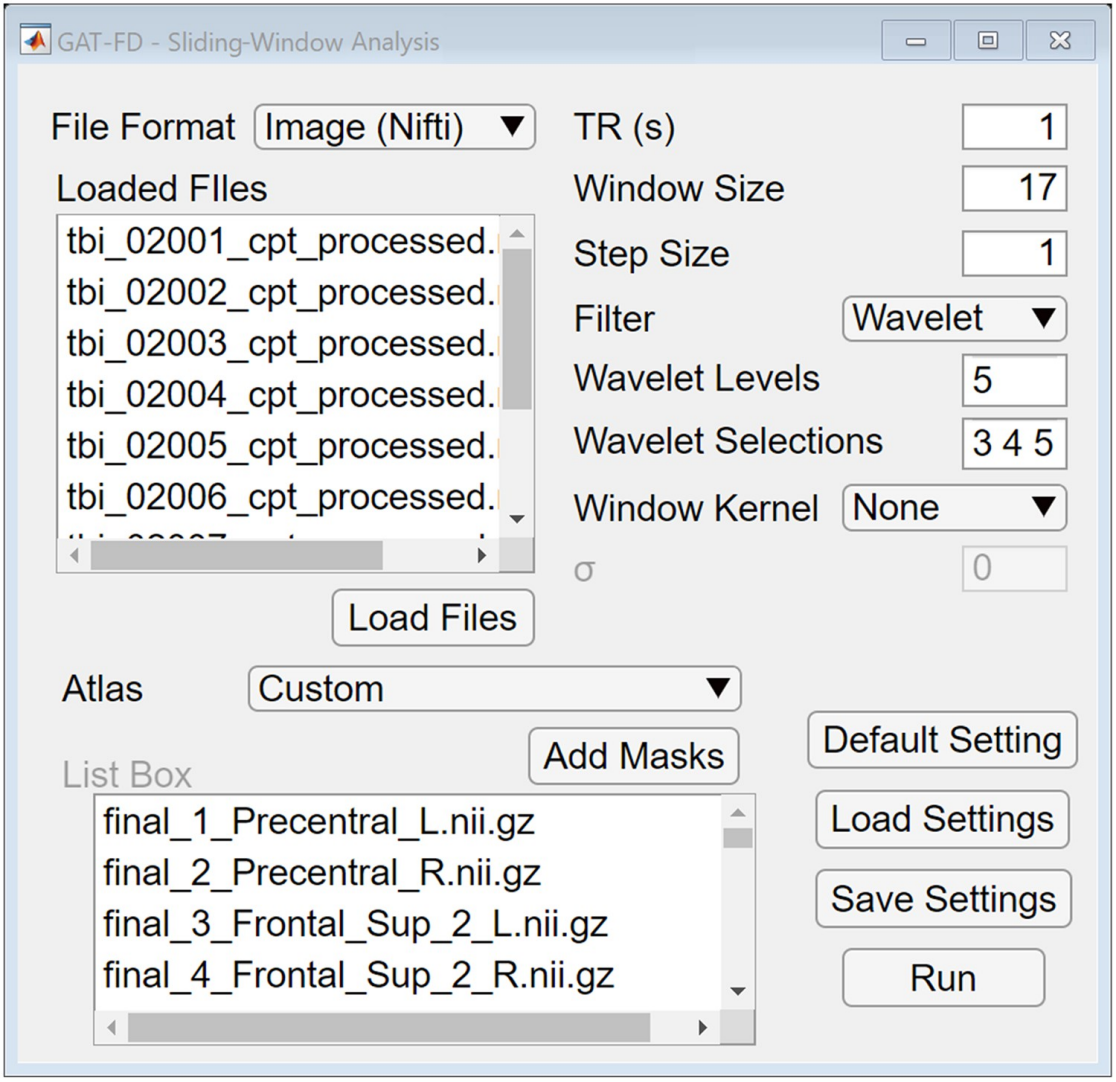
The user interface of sliding-window analysis module. By clicking the “Default Setting” button, users can reset all the parameters to default values. The user specified parameters can be saved and loaded by clicking the “Save/Load Settings” buttons. By clicking the “Run” button, the sliding-window analysis can be performed.

The sliding-window approach is the most common analytical method to explore the network dynamics in fMRI studies [7, 19]. The basic time unit in sliding-window analysis is repetition time (TR), which contains one volume. In this approach, a sliding-window with a fixed length of TRs (called as window size) and a “moving step” (called as step size) along the time series are first defined. Then a FC matrix is constructed, based on the volumes covered by the sliding-window, for each time point that separated by the moving step. The GAT-FD toolbox includes a sliding-window analysis module to extract the activation time series for selected ROIs, perform temporal filtering, apply sliding-window, and construct the FC matrices, as shown in Fig 2. Two options for ROI determination, the customized brain masks and build-in atlas, are available in the module. The toolbox currently provides two build-in atlas, including the automated anatomical labeling (AAL) atlas [20] and Brainnetome atlas [21]. For user-loaded (customized) brain masks and atlas, the MNI space format is required to avoid miscalculations during extraction of blood-oxygen-level-dependent (BOLD) responses. Customized ROIs masks can also be imported with the atlas option set to “Custom”. If customized ROIs were selected, the masks must be in the same coordination space as the input data.

Compared to static FC analysis that uses signals in the entire scan duration to calculate the functional correlations, dynamic FC analysis uses a much smaller window size, which is more sensitive to noises in the data. Therefore, the step of temporal filtering is essential to guarantee the accuracy of detected dynamics of the functional brain network. The sliding-window analysis module of the GAT-FD toolbox includes options of high-, low-, band-pass filter, and wavelet filter to help further minimize the undesired noises. When applying high-, low-, or band-pass filter, the corresponding cut-off values (a lower-limit cut-off value is required for high-pass filter, an upper-limit cut-off value is required for low-pass filter, and both lower- and upper- limits cut-off values are required for band-pass filter) are specified as the inverse of the cut-off frequency, i.e., the unit of second, instead of Hz are implemented here. Wavelet decomposition, as a frequently used tool in task-based fMRI data analysis, increases sensitivity in detecting signal correlation against a noisy background, especially when motion artifacts related spikes occur [22, 23]. If the wavelet filtering function is selected, the sliding-window module will first decompose each activation time series using the maximal overlap discrete wavelet transform (MODWT) with a specific number of levels, and then transfer back with selected levels of coefficients using inverse MODWT. The number of wavelet decomposition levels and the selected wavelet levels need to be specified by the user. The temporal filtering function is optional in this module, given that input data can be already filtered during the pre-processing steps.

In the GAT-FD toolbox, the FC between a pair of ROIs at each step is represented by the Pearson’s correlation coefficient of the BOLD signals within the corresponding sliding-window in the two ROIs. Therefore, the window size and the step size are critical in detecting the desired temporal dynamics during task stimulation-related period when using sliding-window analytical method. The selections of these parameters depend on the task design and the repetition time (TR). Studies have suggested the window size to be larger than 15 TRs, which means at least 15 volumes within a sliding-window, to get reliable estimations of between-region temporal correlations [24–26]. In addition, the window size needs to be smaller than the length of one task block to provide sufficient number of measures on describing mid-task variability [27, 28]. The GAT-FD toolbox also offers an option to utilize gaussian kernel-based sliding-window [7, 10]. Such approach applies a tapered sliding-window with a different weight for each volume involved in the window during the correlation calculation. A series of FC matrices is then generated for each subject with user specified sliding-window parameters. The sliding-window analysis module can also generate connectivity matrix for static functional network by setting the window size equal to the length of the full fMRI task. The sliding-window analysis module is able to process multiple input files at once, to generate one MAT-format output file for each selected subject.

### Temporal inclusive mask generation for block-designed tasks

**Fig 3:**
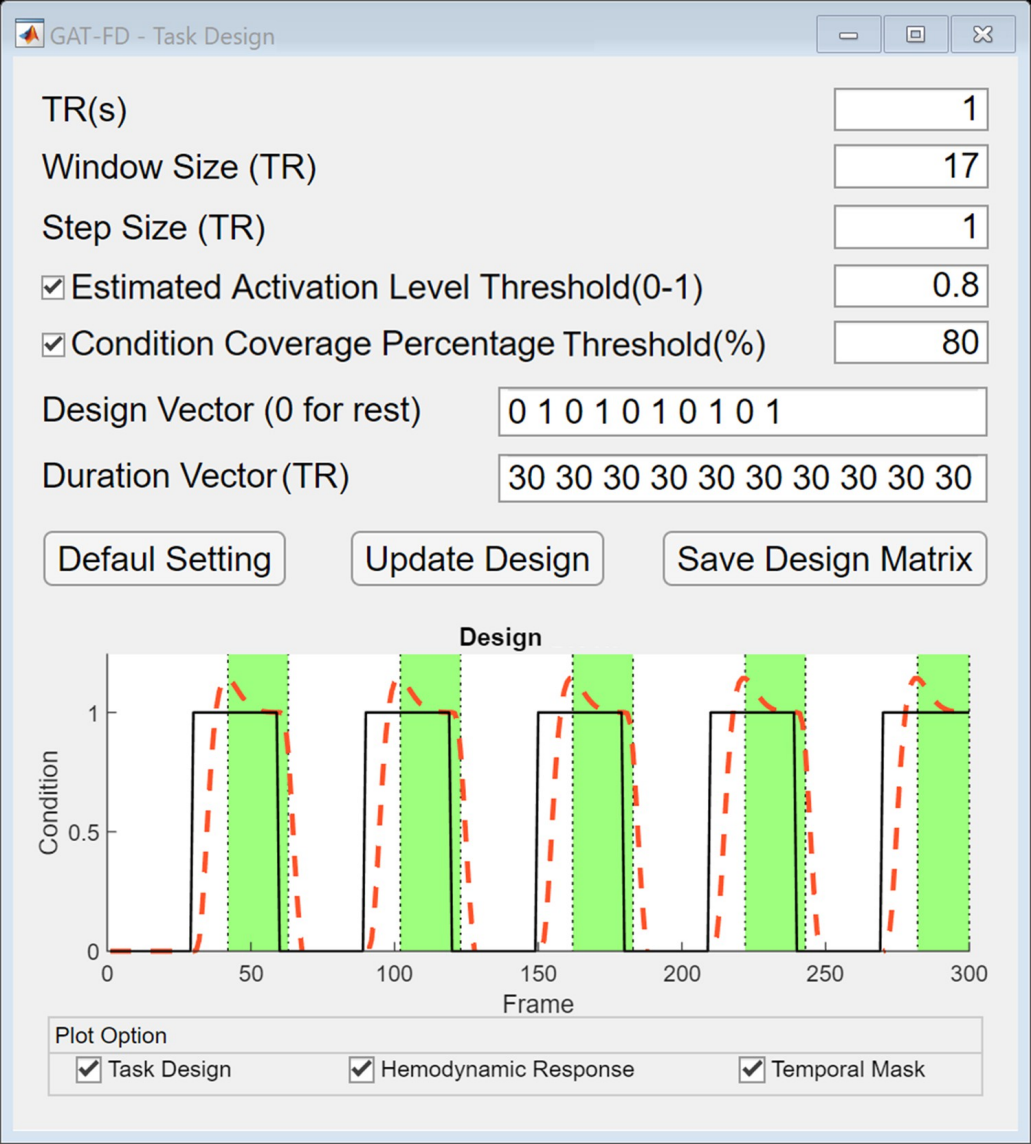
The user interface of task design module. By clicking the “Default Setting” button, users can reset all the parameters to default values. After defining all the parameters, the temporal inclusive mask can be plotted in the bottom by clicking the “Update Design” button. When the “Task Design” plot option is checked, the task design is plotted in solid black line. When the “Hemodynamic Response” plot option is checked, the estimated hemodynamic response is plotted in dashed red line. When the “Temporal Mask” plot option is checked, the time points selected by the temporal inclusive mask are shown in green areas. The temporal inclusive mask file needs to be saved by clicking on the “Save Design Matrix” button.

Studies have found that the pattern of pairwise FC varies between the rest and task conditions during fMRI [29, 30]. To map the task-specific (or rest-specific) FC matrices in the sliding-window analysis module, the temporal inclusive mask generated from the task design module of the GAT-FD toolbox can be needed. To determine the temporal inclusive mask, two thresholding methods, estimated activation level thresholding and condition coverage percentage thresholding, are provided (Fig 3). Users can choose to use either or both methods to generate their study-specific masks by checking the box of each function. If both boxes are checked, logic AND will be implemented to the two masks for the final output of the temporal inclusive mask. The estimated activation level thresholding method is based on the estimated hemodynamic responses which are internally estimated in the module by convolving the task design with the hemodynamic response function in Statistical Parametric Mapping (SPM) toolbox [31]. If the box for estimated activation level method is checked and the threshold is provided by the user (or by using default value), the time points with the estimated activation magnitude (ranged from 0 to 1, with 1 representing the maximum estimated response for a single stimulus) higher than the user defined threshold are included in the temporal inclusive mask. By checking the box of condition coverage percentage thresholding method, selecting the condition type (1 for task and 0 for rest), and defining the percentage threshold (X%), time points with at least X% of their associated sliding windows under the selected task condition (according to the task design) will be inclusive in the mask. The default settings for these two parameters are 0.8 and 80, respectively. The output of this module is a temporal inclusive mask which contains the indices of the task stimulation-related study-specific FC matrices. In addition, users can manually define the temporal inclusive mask.

### Characteristics of the topological dynamics of the functional brain network

To characterize the topological dynamics of the functional brain network, the first step is to binarize the involved FC matrices. The network analysis module of the GAT-FD toolbox provides multiple thresholding methods for FC matrix binarization, including absolute thresholding, proportional thresholding, and wiring cost thresholding. The absolute thresholding method applies the same user specified correlation coefficient threshold (ranging from -1 to 1) to all connectivity matrices. The proportional thresholding method defines the threshold relative to the maximum correlation coefficient in each FC matrix with user specified proportion, ranging from 0 to 1. The wiring cost thresholding method preserves the top connections (with user specified cost threshold) in each FC matrix. The cost of a network is defined as the number of existing connections divided by the number of all possible connections, which ranged from 0 to 1, representing the top 0% to 100% connections, respectively. The toolbox also includes the option of using absolute value of the correlation coefficient for thresholding.

Estimations of topological properties at both the global- and nodal-level are provided in the toolbox. The global-level properties include network global efficiency, network local efficiency, network clustering coefficient, network average degree, characteristic path length, normalized clustering coefficient, normalized path length, small world coefficient, transitivity coefficient, assortativity coefficient, and modularity coefficient. The nodal-level properties include nodal global efficiency, nodal local efficiency, nodal clustering coefficient, nodal degree, and betweenness centrality. These topological properties are calculated from the binarized FC matrices.

The network global efficiency, *E*_*glob*_(*G*), is a metric of the structural network integration that reflects the ability of information transferring across distributed brain areas [32]. It is defined as:

$$E_{glob}(G)=\frac{1}{n(n-1)}\sum_{i,j\in G,j\neq i}\frac{1}{d_{ij}}$$ (1)

where *n* is the number of nodes in the network, and *d*_*ij*_ is the inverse of the shortest path length (number of edges) between node *i* and *j*.

The network local efficiency, *E*_*network*−*loc*_(*G*), estimates the network segregation and represents the fault tolerance level of the network [32], which is defined as:

$$E_{network-loc}(G)=\frac{1}{n}\sum_{i\in G}E_{glob}(G_{i})$$ (2)

where *G*_*i*_ is the subnetwork that consists of all neighbor nodes of node *i*, and the global efficiency of subnetwork *G*_*i*_ is calculated using equation Eq (1).

The network averaged degree is the average number of neighbors (degree) of all nodes in the network, represents the overall connectivity strength in the network.

The network clustering coefficient, *C*(*G*), is the mean clustering coefficient of all nodes in the network, which represent the fraction of the node’s neighbors that are also neighbors of each other [33]. It is defined as:

$$C(G)=\frac{1}{n}\sum_{i\in G}\frac{1}{k_{i}(k_{i}-1)}\times \sum_{j,h\in G_{i}}{\left(a_{ij}a_{ih}a_{jh}\right)}^{1/3}$$ (3)

where *a*_*ij*_ is the connection between node *i* and *j* (1 for connected and 0 for not connected), and *k*_*i*_ is the number of neighbors of node *i*.

The characteristic path length, *L*(*G*), is the average length of the shortest path between all node-pairs in the network [33]. It is an alternative of network global efficiency, which is defined as:

$$L(G)=\frac{1}{n(n-1)}\sum_{i,j\in G,j\neq i}d_{ij}$$ (4)

The normalized clustering coefficient, *C*_*norm*_ or *γ*, is the ratio between the network clustering coefficient of the current network and similar random networks [33]. It is defined as:

$$C_{norm}=C_{network}/C_{rand}$$ (5)

where *C*_*rand*_ is the average of network clustering coefficient of multiple random networks (20 random networks are used in the GAT-FD toolbox) that have the same number of edges and nodes as the current network.

The normalized path length, *L*_*norm*_ or *λ*, is the ratio between the characteristic path length of the current network and similar random networks [33]. It is defined as:

$$L_{norm}=L_{network}/L_{rand}$$ (6)

where *L*_*rand*_ is the average of characteristic path length of multiple random networks (20 random networks are used in the GAT-FD toolbox) that have the same number of edges and nodes as the current network.

The small world coefficient, *S* or *σ*, is the ratio between normalized clustering coefficient and normalized path length, which is defined as:

$$S=C_{norm}/L_{norm}$$ (7)

The transitivity coefficient is a similar measure as network clustering coefficient, which is the fraction of closed triplets in all possible triplets of a network [34].

The assortativity coefficient characterizes the connectivity similarity of all connected node-pairs in the network, which is the correlation coefficient between the degrees of nodes on both sides of all edges [35].

The modularity coefficient is the quantitative measure of the ability to subdivide a network into clearly separated modules [36].

The nodal global efficiency, *E*_*nodal*_(*i*), is a measure of nodal communication capacity of a node with all other nodes in the network, which is defined as:

$$E_{nodal}(i)=\frac{1}{n-1}\sum_{j\in N,j\neq i}\frac{1}{d_{ij}}$$ (8)

The nodal local efficiency of node *i* represents the robustness and integration of the subnetwork it belongs, which was defined as the network global efficiency of the subnetwork that consists of all the neighbors of *i*.

The nodal clustering coefficient, *C*(*i*), describes the likelihood of whether the neighboring nodes of node *i* are interconnected with each other [33]. It is defined as:

$$C(i)=\frac{1}{k_{i}(k_{i}-1)}\sum_{j,h\in N_{i}}{\left(a_{ij}a_{ih}a_{jh}\right)}^{1/3}$$ (9)

The nodal degree is the number of neighbors of a specific node. It represents the network connection strength of the node.

The betweenness centrality, *B*(*i*), attempts to measure the ability for one node to bridge indirectly connected nodes [37]. It is defined as:

$$B(i)=\frac{1}{\left(n-1)(n-2\right)}\sum_{j,k\in N,j\neq k}\frac{p(i|j,k)}{P(j,k)}$$ (10)

where *j*, *k* were node pairs in the network. *p*(*i*|*j*, *k*) was whether the shortest path between node *j* and node *k* passes through node *i*. *P*(*j*, *k*) was the total number of unique shortest path between node *j* and node *k*.

**Fig 4:**
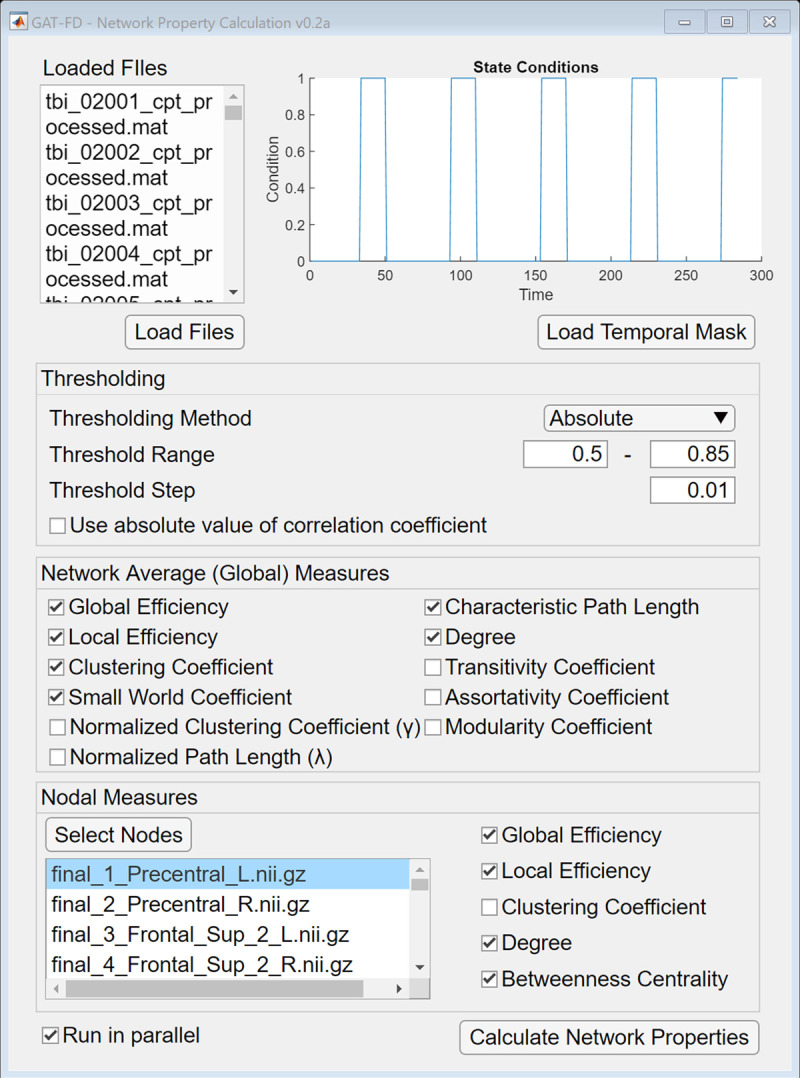
The user interface of network analysis module. The selected timepoints are displayed at the top right corner after loading the temporal mask file. If the “Use absolute value of correlation coefficient” is checked, all the negative correlation coefficients in the connectivity matrices are converted to positive value before thresholding. By clicking the “Calculate Network Properties” button, the network analysis is performed with user specified thresholding parameters and user selected topological measures.

The temporal dynamics of a global- or nodal-level topological property are measured using the variance of this property over the selected time range (or multiple time ranges defined in Section 2.4) during the entire scan period. The variance is used to characterize the stability of a topological property, which is defined as:

$$V=\frac{1}{N-1}\sum_{i=1}^{N}{\left|A_{i}-\mu \right|}^{2}$$ (11)

where N is the number of time points, *A*_*i*_ is the network topological property at that time point, and *μ* is the mean of the network topological property over the selected time points. The GAT-FD toolbox utilizes functions from the brain connectivity toolbox for network construction and topological features calculation [38]. The calculations are time consuming, therefore, parallel computing option in this module is supported if the MATLAB parallel toolbox is installed. The detailed configurable parameters are shown in Fig 4.

### Result display

**Fig 5:**
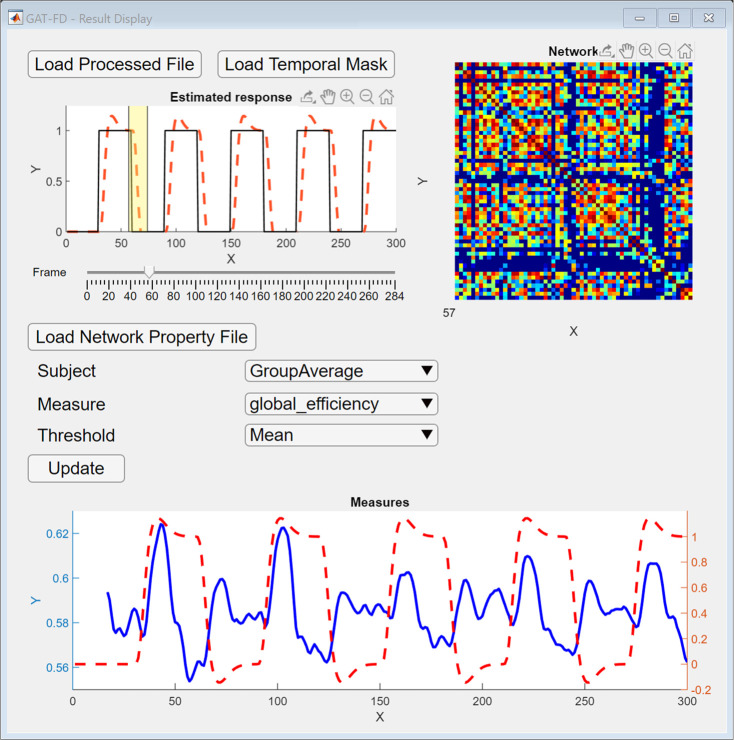
The user interface of result display module. After loading the generated connectivity matrices file and temporal mask file, the functional connectivity matrix can be displayed at top right conner by selecting the desired sliding-window step using the “Frame” slider. After loading the generated network properties file, the network topological properties for different subjects and different threshold values can be displayed at the bottom by clicking the “Update” button.

Result display module provides convenient features to visually check the calculated results from all previous modules, as shown in Fig 5. The constructed connectivity matrices for each sliding-window can be checked for any abnormal conditions. Task design can be loaded and displayed to provide a visual inspection for sliding-window selection. The results from the network analysis module can also be loaded in the display module. The global-level topological properties selected in the network analysis module can be plotted at individual level or group level, along with the estimated hemodynamic response. The nodal-level topological properties are stored as MATLAB matrix in the result file. Due to the size of the results matrix, the nodal-level topological properties cannot be plotted and can only be accessed by loading the result file into MATLAB.

## Illustration

### Data

Task-based fMRI data from 40 typically developing children (male/female: 22/18) were involved in the validation and illustration of the GAT-FD toolbox. All subjects were 11 to 16 years old, right-handed according to the Edinburgh Handedness Inventory [39], within or post puberty based on the parent version of Carskadon and Acebo’s rating scale [40], and had full scale IQ≥ 80 estimated by the Wechsler Abbreviated Scale of Intelligence II (WASI-II) [41]. None of the subjects reported a history or current diagnoses of neurological and psychiatric disorders, chronic medial illnesses, or learning disabilities. None of them had been taking stimulant or non-stimulant medications within the past 3 months prior to the study visit that might impact the brain activations during fMRI data acquisition. The study received institutional review board approval at the New Jersey Institute of Technology and Saint Peter’s University Hospital. Prior the study, all the participants and their parents or legal guardians provided written informed assents and consents, respectively.

Each participant performed a block-design visual sustained attention task (VSAT) during the fMRI scan. The VSAT included 5 task blocks interleaved by 5 rest blocks, each was 30 seconds. Detailed descriptions of the task were provided in our previous studies [42, 43]. The fMRI data were collected using a 3-Tesla Siemens TRIO (Siemens Medical Systems, Germany) scanner with a whole brain gradient echo-planar sequence (voxel size = 1.5 mm × 1.5 mm × 2.0 mm, TR = 1000 ms, echo time = 28.8 ms, and field of view = 208 mm, slice thickness = 2.0 mm).

### Preprocessing

The preprocessing steps were performed using FEAT toolbox in FMRIB’s Software Library [44]. Each raw data was first corrected for slice timing and motion artifacts, using sinc interpolation and rigid-body transformation, respectively. Then brain extraction was performed to remove non-brain tissues using the averaged fMRI data. Spatial smoothing was performed with a 5-mm full-width at half maximum gaussian kernel to improve the signal-to-noise ratio. The signal intensity was then normalized for each slice. Then, a high-pass temporal filter of 1/75 Hz was applied to remove low frequency noises. Finally, linear registration was performed to the MNI template with a voxel size of 2 × 2 × 2 mm^3^.

The group average activation map within the study cohort was calculated and parcellated according to the AAL atlas [20]. Within each parcellated brain regions that contain at least 100 significantly activated voxels (Z ≥ 2.3 after cluster correction for multiple comparisons), a spherical ROI with the radius of 5mm and centered at the regional maximum of the activated cluster was generated in the MNI space. A total of 59 ROIs (nodes for the to be constructed functional brain networks) were generated and mapped back to each pre-processed fMRI data to construct the dynamic functional networks.

### The GAT-FD-based processes

For data from each subject, wavelet-based temporal filtering was first performed on the time series of each ROI, using the 5-level wavelet transformation. The level 3,4, and 5, corresponding to frequency band of 0.015–0.124 Hz, were then used to reconstruct the time series for each ROI. This frequency band has been demonstrated to contain most task-related hemodynamic information [4, 42, 45]. Then, the sliding-window analysis was performed with the window size of 17 TRs and the step size of 1 TR. Such window size and step size were suggested to be able to generate reliable temporal correlation coefficient (the FC measure) within each sliding step and offer enough sliding steps that allows for estimations of variability of the FC during the task stimulation period [26–28]. A total of 284 connectivity matrices were then generated for each subject.

As an example of illustrating the characterization the task stimulation-related dynamics of the network properties, a temporal inclusive mask was generated based on the blocked design of the task. The condition vector was set as “0 1 0 1 0 1 0 1 0 1”, where 0 was for a rest block and 1 for a task block. The duration vector for each block was converted from second to TR and set as “30 30 30 30 30 30 30 30 30 30”. The estimated activation level threshold was set as 0.8, and the task condition coverage percentage threshold was set as 80%, as suggested by default. By implementing this temporal inclusive mask, a total of 79 FC matrices, which were generated in the sliding-window analysis module, were marked as task stimulation-related.

In the network property analysis module, the absolute thresholding method was implemented for the binarization of the 284 FC matrices generated in the sliding-window analysis module. The range of correlation coefficient threshold was set as from 0.5 to 0.85 with a step size of 0.01. Such threshold range was calculated based on the cost-range of the functional network that satisfied the small-world network assumption [33, 46]. Then, the global- and nodal-level topological properties of each of the 284 functional brain networks were calculated. In each subject, the variance of each topological property was calculated over the 284 time points in the overall scan duration and over the 79 task stimulation-related time points based on the generated temporal mask. The group average of network topological properties was then calculated. In addition, variances of the network properties calculated over the full scan duration were compared with those calculated based on the generated temporal mask, using paired sample t-test, with a threshold of significance at α≤ 0.05.

## Results and discussion

**Fig 6:**
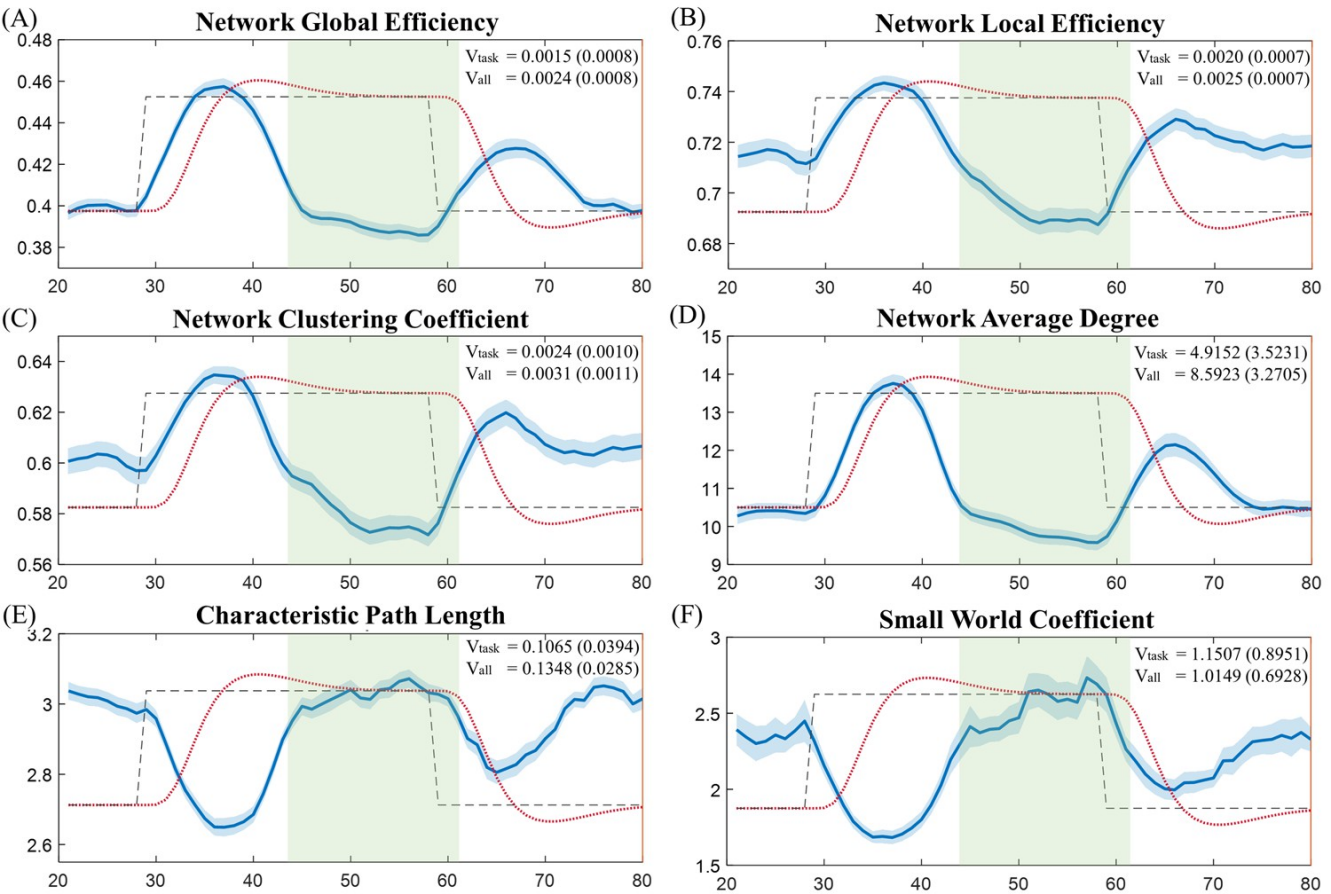
Group averages of the network global properties. Each global property was averaged over the first 4 task blocks (the solid blue line). The light blue shadowed area represents the standard error of the mean of each property. The task condition is plotted using dashed black line. The estimated hemodynamic response over the testing period is plotted using dotted red line. The task stimulation-related period defined using the task design module is shadowed in light green. (A) Network global efficiency. (B) Network local efficiency. (C) Network clustering coefficient. (D) Network average degree. (E) Characteristic path length. (F) Small world coefficient. (Vtask: Variance of the topological properties over the task-related period. Vall: Variance of the topological properties over the entire scan duration.).

As an example of visualization, Fig 6 illustrated group averages of the global-level topological properties generated from our testing samples, including the network global efficiency, network local efficiency, network clustering coefficient, network average degree, characteristic path length, and small world coefficient, respectively. We observed decreased characteristic path length and increased network global efficiency, network local efficiency, network clustering coefficient, and network average degree in the functional brain network during both rest-to-task and task-to-rest transition periods, while a relatively steady state of these topological properties in the middle of the task blocks. Such pattern of task stimulation-related FC dynamics has also been observed in other studies [26, 47–49]. The small world coefficient of the functional network over all time points were within the range of 1.6 to 2.8, as shown in Fig 6F, which has been observed as a typical small world coefficient range for common neural networks in both humans and animals [50, 51]. In addition, all the global topological properties showed significantly lower variances during the task stimulation-related period covered by the temporal mask, when compared to those estimated over the entire scan duration (the network global efficiency (t = 2.282, *p* = 0.028), network local efficiency (t = 2.223, *p* = 0.032), network clustering coefficient (t = 2.235, *p* = 0.031), network averaged degree (t = 2.529, *p* = 0.016), and characteristic path length (t = 3.989, *p*<0.001)). Indeed, superior topological stability of the functional brain network during task performance relative to that during resting state has also been reported by other studies [52, 53].

**Fig 7:**
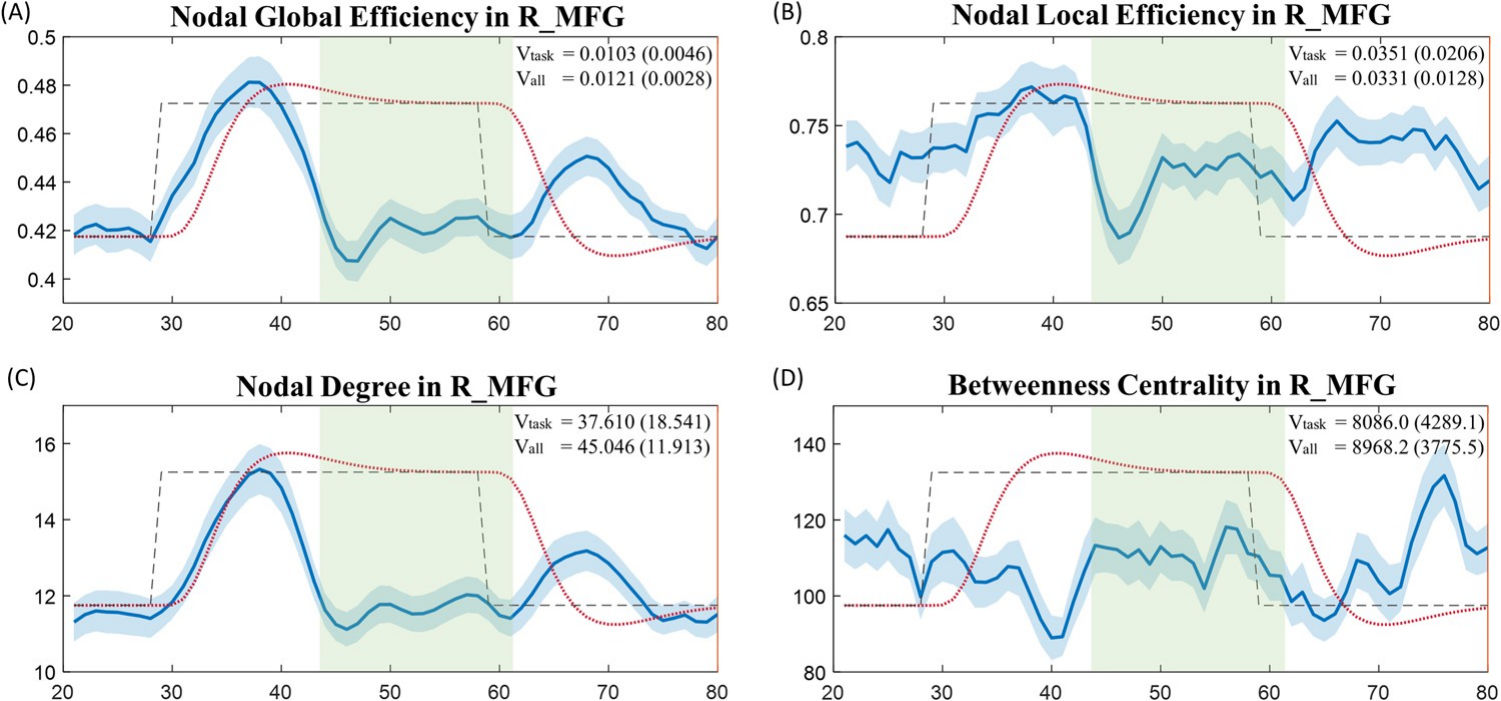
Group average of the nodal topological properties in right middle frontal gyrus. Each nodal property was averaged over the first 4 task blocks (the solid blue line). The light blue shadowed area represents the standard error of the mean of each property. The task condition is plotted using dashed black line. The estimated hemodynamic response over the testing period is plotted using dotted red line. The task stimulation-related period defined using the task design module is shadowed in light green. (A) Nodal global efficiency. (B) Nodal local efficiency. (C) Nodal degree. (D) Betweenness centrality. (R_MFG: Right Middle Frontal Gyrus. Vtask: Variance of the topological properties over the task stimulation-related period. Vall: Variance of the topological properties over the entire scan duration.).

To illustrate the results of nodal-level topological properties and their dynamics, Fig 7 included group averages of the nodal global efficiency, nodal local efficiency, nodal degree, and betweenness centrality in the node of right middle frontal gyrus (MFG). Right MFG has been found to serve as a critical component in the visual sustained attention pathways and play key role in the dorsal and ventral attention networks [54–56]. We observed that, relative to those over the entire scan duration, the variances of the nodal global efficiency and nodal degree of the right MFG were significantly reduced during the task stimulation-related (the nodal global efficiency (t = 2.269, *p* = 0.026) and nodal degree (t = 2.289, *p* = 0.024)), which suggests strong functional stability and significant involvement of the right MFG during sustained visual attention processing. These results are consistent with findings from previous fMRI studies that showed significant activations in right MFG during attention-related tasks [56–58].

Compared to the existing toolboxes that provide dynamic network analysis, GAT-FD toolbox is able to 1) bring extra flexibility in defining task-related time points for dynamic analysis; 2) integrate the calculation of network topological properties with dynamic network construction. On the bases of the initial pipelines provided in the current version of the GAT-FD toolbox, future work will focus on developing and including more alternative analytical techniques for characterizing the dynamics of FC and network properties.

## Conclusion

In this study, we introduced an integrative MATLAB toolbox, GAT-FD, for analyzing the task-related dynamics of FC and topological properties of the functional brain networks for sensory and cognitive processes during task-based fMRI, especially for block-designed data. All the involved functions have been tested and validated using data collected from human subjects during task-based fMRI. The results demonstrated that the GAT-FD allows for effective and quantitative evaluations of the functional network properties and their dynamics during the entire fMRI scan or user-specified periods. The GAT-FD toolbox and user manual are freely available at https://www.nitrc.org/projects/gat_fd and https://centers.njit.edu/cnnl/gat_fd/.
